# Assessing the effect of prophylactic ankle taping on ankle and knee biomechanics during landing tasks in healthy individuals: A cross-sectional observational study

**DOI:** 10.1590/1516-3180.2022.0548.R1.10032023

**Published:** 2023-07-31

**Authors:** Carlos Romero-Morales, Ana Matilde-Cruz, María García-Arrabe, Felix Higes-Núñez, Alexandre Días Lópes, Sergio Jiménez Saiz, Helios Pareja-Galeano, Daniel López-López

**Affiliations:** I PT, PhD, MSc. Senior Lecturer, Faculty of Sport Sciences, Universidad Europea de Madrid, Villaviciosa de Odón, Madrid, Spain.; II MSc. Lecturer, Faculty of Sport Sciences, Universidad Europea de Madrid, Villaviciosa de Odón, Madrid, Spain.; III PhD. Lecturer, Faculty of Sport Sciences, Universidad Europea de Madrid, Villaviciosa de Odón, Madrid, Spain.; IV MSc. Lecturer, Faculty of Sport Sciences, Universidad Europea de Madrid, Villaviciosa de Odón, Madrid, Spain.; V PT, PhD. Clinical Professor, Department of Physical Therapy, Movement and Rehabilitation Sciences, Northeastern University, Boston, Massachusetts, United States; VI PhD. Full Professor, Centre for Sport Studies, Universidad Rey Juan Carlos, Madrid, Spain.; VII PhD. Lecturer, Department of Physical Education, Sport and Human Movement, Universidad Autónoma de Madrid, Madrid, Spain.; VIII PhD. Senior Lecturer. Research, Health and Podiatry Group. Department of Health Sciences. Faculty of Nursing and Podiatry. Industrial Campus of Ferrol. Universidade da Coruña, Spain.

**Keywords:** Ankle injuries, Biomechanical phenomena, Kinetics, Hopping training, Kinematics, Biomechanics

## Abstract

**BACKGROUND:**

Current research supports the fact that prophylactic ankle taping (AT) is effective in preventing ankle injuries in amateur and elite sports athletes.

**OBJECTIVE:**

This study aimed to investigate the effect of AT on balance, knee valgus during drop jump and single-leg countermovement jump (SL-CMJ) landings, and ankle range of motion (ROM) restriction in healthy participants.

**DESIGN AND SETTING:**

A cross-sectional observational study was conducted at the Universidad Europea de Madrid, Madrid, Spain.

**METHODS:**

Participants: Thirty-nine healthy individuals participated in this study and performed the movements under two conditions (with and without tape). Outcome measurements: ankle ROM, balance, SL-CMJ height, flight time, ground time, and knee valgus. Before any intervention, a random process was developed with a 1:1 allocation ratio, and the participants were assigned to groups A (tape-no tape) and B (no tape-tape).

**RESULTS:**

Significant differences between tape and no-tape moments were observed for drop jump knee valgus flexion (P = 0.007), with an increase in knee valgus in participants with ankle taping. Similarly, the Y-balance testshowed a significant decrease in all variables (P = 0.001 and), ankle dorsiflexion (P = 0.001) in participants with ankle taping.

**CONCLUSIONS:**

AT is effective for immediate ankle ROM restriction. However, an increase in knee valgus during drop jump task and a decrease in lower limb balance were observed during drop jump task. Based on these results, it can be concluded that AT application in healthy individuals should not be recommended as it results in increase in injury risk factors.

## INTRODUCTION

Current research supports the fact that prophylactic ankle taping (AT) is useful in preventing ankle injuries in amateur and elite sports athletes. It provides extra stabilization of the ankle joint.^1^ The primary strength of AT is limitation in the range of motion (ROM) of tibiotalar and subtalar joints, which results in an increase in the proprioceptive outputs.^2^ Several studies have reported the efficacy of prophylactic approaches with rigid tapes and bracing in protecting the soft tissues and ligaments in maximal stress situations (e.g. jumps, landings, change-of-directions).^3,4^ AT has been employed in sports and non-sports populations in rehabilitation and prevention to reduce the incidence of ankle sprain injuries that commonly occur during training, amateur or professional competition. The effects of rigid or semi-rigid tape approaches not only influences ankle joint restriction, but also has effect on other movements. For example, electromyography assessment reported a decrease in the peroneus contraction time and a decrease in the average eversion and inversion velocity times.^5,6^ Other undesirable effects of ankle bandages have been reported, such as a decrease in jump performance in athletes or dermatologic manifestations.^7,8^

Extensive research has demonstrated the efficacy of AT in ROM restriction and injury prevention. Pederson et al. reported the prophylactic approach of AT in ankle joint fixation among Rugby players.^9^ In the context of eversion-inversion limitation movements, Callaghan et al. showed the benefits of AT in non-weight bearing positions.^10^ Several systematic reviews support the use of rigid and elastic bandages in individuals with ankle sprain history for prevention and rehabilitation.^11,12^

Elite and amateur sports environments improve prevention and rehabilitation programs to decrease sports injuries. For example, the incidence rate of ankle sprain injury reported among basketball players is 3.85 per 1,000 individuals, and the primary cause of these injuries is the landing phase of jump movement.^13^ Sport medicine doctors and medical staff focus on lower limb biomechanics to decrease the injury ratios.

Despite the evidence of reduction in the likelihood and severity of ankle sprain injury, restriction of normal foot and ankle biomechanics may increase the risk of injury to proximal joints, such as the knee. Previous studies on ski-boots have reported that these provide excellent ankle joint protection during sport performance; however, they have been associated with lower limb biomechanical disturbances, such as knee injuries.^14^ Knee abduction motion, generally known as knee valgus, has been described as a factor associated with increased load on the knee joints and potential anterior cruciate ligament (ACL) injury during landing and change-of-direction biomechanics.^15–18^ In this context, several authors have reported that knee abduction and medial movements during landing tasks were predisposing factors for development of ACL injury or patellofemoral pain in athletes, especially among females.^17–19^

Santos et al. delineated that AT was less rigid than a ski-boot. Thus, alterations in the ankle joint kinematics were observed with rigid tape and bracing during simple tasks.^20^ Similarly, studies have reported that valgus movements and internal rotation of tibia play an important role in ACL injuries. For example, Stoffel tel al. reported a reduction of 5 Nm in knee internal rotation during running/sidestepping tasks in individuals with AT compared to controls.^1^

## OBJECTIVE

This study aimed to investigate the effect of AT on balance, knee valgus during drop jump and single-leg countermovement jump (SL-CMJ) landings, and ankle ROM restriction in healthy participants. It was hypothesized that AT would be effective in restricting ankle ROM. However, it could result in a decrease in balance and an increase in knee-ankle valgus during landing in drop jumps and SL-CMJ tasks.

## METHODS

### Design and sample

This cross-sectional, descriptive, single-blinded, observational study was conducted in accordance with the Strengthening the Reporting of Observational Studies in Epidemiology (STROBE) guidelines between February 2022 and May 2022 at the Research Lab of Universidad Europea, Madrid, Spain.

Thirty-nine healthy individuals were recruited for the study from the Universidad Europea Sport Facilities. Participants were excluded if they presented with any musculoskeletal condition requiring treatment during a period of three months prior to testing. Individuals with dermatologic disorders or tape allergy, and those who underwent lower-limb surgery or had foot orthoses were also excluded from the study.

### Ethical considerations

This study was approved by the Universidad Europea Research Ethics Committee (CIPI/213006.97; Dated: December 16, 2021). Written informed consent was obtained from all the participants before commencement of the study. All the procedures in this study were performed in accordance with the tenets of Declaration of Helsinki.

### Sample size

According to Williams et al.,^21^ a convenience sample of 21 participants was considered sufficient to evaluate the effectiveness of AT on knee biomechanics during jumping and landing tasks. Finally, a total sample of 39 participants was recruited for the present study.

### Study Procedure

Before the assessment, basic anthropometric measures (height, weight, and body mass index) were recorded using a calibrated device, and the participants were instructed to complete a questionnaire to ensure that the study inclusion criteria were met.

### Randomization and blinding

Before any intervention, a random process was developed using the free software system (randomization.org) with a 1:1 allocation ratio, and the participants were assigned to groups A (assessments with tape assessments with no tape) and B (assessments with no tape- assessments with tape). All participants wore a pair of long socks thatidwhich do not allow the rater to know whether they were taped.

### Ankle taping

The AT procedure was developed by an experimental physical therapist with more than five years of experience in taping in an elite sport environment. The ankles of the participants were covered with pre-wrap before the taping procedure in accordance with the Sports Medicine guidelines for taping methods.^22^ AT was performed with a standard 38-mm self-adhesive tape starting with two anchor strips around the leg 10 cm above the malleoli. The next step consisted of two strips being placed from the medial side of the anchor tape to the lateral side with the foot in a neutral position.^21^ The “figure sixes” focusing on the subtalar joint were performed with an initial strip onto medial anchor thorough the plantar aspect of the foot attached onto the medial anchor. To complete the AT procedure, the therapist covered all free ends and spaces with tape.^21^

### Movement tasks

All jump trials were assessed by the same evaluator using standardized verbal commands. Before the measurements, each participant was instructed to perform a 10-minute warm-up session. Subsequently, for the drop-jump test, each participant jumped from a 30 cm box, with hands placed on the hips. Participants were instructed to: “jump up as fast as possible after contact and try to jump as high as possible with one leg”.^23^ To initiate the drop, the participants were instructed to not jump out of the platform, rather just step out with one foot. Two jumps were performed, and the better result achieved for each jump were registered for the analyses. For SL-CMJ, participants were instructed to place one foot on the ground and the free leg behind at approximately 80-90º with their hand on the iliac crests, and then jump as high as possible.^24^ In the same way, two trials were performed and recorded, and the highest jump was analyzed.

### Outcome measurements

**Figure 1:**
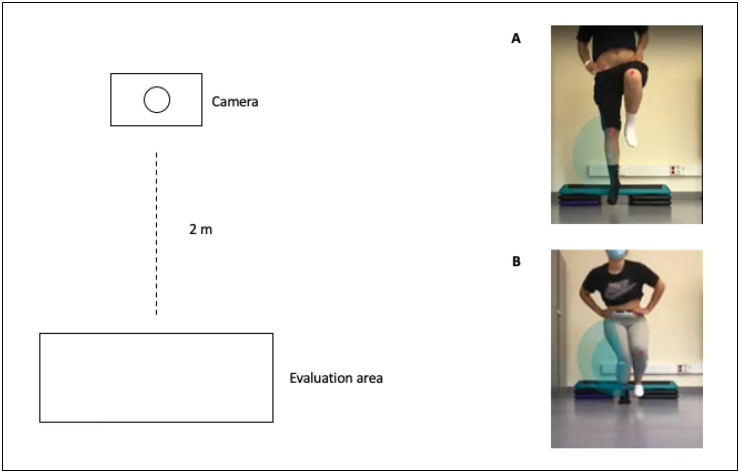
Drop-jump and countermovement jump assessments.

Three-dimensional (3D) motion capture tools have been considered the “gold standard” for assessment and quantification of human movement.^25^ Hanzlikova et al. reported that 3D systems were reliable in evaluating the multi-planar kinematics of the knee joint during functional tasks (e.g. landings, change-of-direction, cutting maneuvers).^26^ However, due to the increased cost factor and difficulty in accessing the 3D systems, several two-dimensional (2D) methods have been developed and validated.^27–30^ Irawan et al. reported that 2D tools for kinematics assessment was a reliable, unexpensive, and easy to use method that can be used in the clinical and research fields to evaluate knee valgus movement based on frontal plane projection angle during drop-jump and single leg landings.^25^ The combination of smartphones-Kinovea has been proven to be a valid and reliable instrument for evaluation of joint kinematics and jump performances in different populations.^31^ Therefore, in the present study, the iPhone 12 camera with 18 mm lens was used and it was positioned 2 m away from the evaluation zone. No zooming effect was applied at any time to standardize the procedure for all participants. All videos were imported into the freeware motion analysis Kinovea software (GPLv2 licence) [this software was created via non-profit collaboration of several researchers worldwide]. Kinovea is a free 2D motion analysis software that can be used to assess kinematic parameters. Several authors have used Kinovea to evaluate running and vertical jump’s or landings among athletes.^32,33^ To assess knee valgus movement, the angle between the line from the anterior superior iliac spine (ASIS) to the middle of the patella and the line from the ASIS to the center of the ankle joints^25^ on the frontal plane was measured. Although Kinovea allows analysis of kinematic parameters without any skin markers, these markers were placed on the ASIS and in the middle of the patella to improve the reliability of the evaluations.^34^ One physical therapist with more than five years of experience in human motion analyses measured knee valgus angle in the frontal plane projection angle which resulted in the development of drop-jump test and SL-CMJ with Kinovea software (Figure 1).

Kinovea software was used to measure flight time, ground time contact, and jump height. Then, the first frame in which the foot left the floor completely (take-off phase) and the first frame in which the foot touched the floor again (landing phase) were employed to calculate the flight time and ground contact time. Flight times from the jump test by identifying takeoff and landing phases were used to calculate jump height using the equation described by Bosco et al.^35^

Y- balance test (YBT) was performed to assess balance. It consisted of three lines attached to the floor in the anterior, posteromedial, and posterolateral directions. Following the guidelines of Plisky et al., posterior lines were placed 135 from the anterior line, with 45 between the posterior lines.^36^ Prior to the test, participants viewed a demonstration made by the rater to familiarize themselves with the process and practiced six trials on each leg in the three directions. Participants were instructed to stand barefoot at the center of the “Y” and each participant had to maintain a single-leg posture of the target limb and try to reach the maximum distance in every three direction. Hands were placed at the iliac crest, and the stance heel was in contact with the ground.^36^ If the participants did not follow the instructions or any criteria were violated, the trial was repeated.

Maximal ankle dorsiflexion ROM was assessed using the valid and reliable *My Rom app* (Madrid, Spain, v.3.0.4) for the iPhone.^37^ Participants were instructed to be in a weight-bearing lunge position and the device was placed under the tibial tuberosity. Each participant developed a maximal dorsiflexion of the ankle joint that was valued, and the application automatically reported the dorsiflexion angle and ankle asymmetry.

All the outcome measurements were carried out by the same investigator.

### Statistical analysis

SPSS software (version 23.0; IBM SPSS Statistics, Armonk, IBM Corp, United States) was used for statistical analyses. Shapiro-Wilk test was used to assess the normality of data distribution. Student’s t-test and Mann-Whitney U test were used to check the differences between the groups (tape-no-tape and male-female comparisons) for parametric and non-parametric data, respectively. In addition, Levene’s test was used to assess the equality of variances. The intraclass correlation coefficient (ICC) was calculated to evaluate the intra-rater reliability of all measurements. The level of significance was set at P < 0.02 with an α error of 0.05 (95% confidence interval) and a desired power of 80% (β error of 0.2).

## RESULTS

Sociodemographic data showed differences in height and weight between male and female participants, (Table 1). As shown in Table 2, significant differences were observed between tape and no tape movements in terms of drop jump knee valgus flexion (P = 0.007), with an increase in knee valgus in participants with AT. Similarly, the YBT and ROM tests showed a significant decrease in medial (P = 0.001), lateral (P = 0.001), and anterior (P = 0.001) ankle dorsiflexion (P = 0.001) in participants with AT. Ankle dorsiflexion asymmetry also increased between ankles with and without taping (P = 0.001). As shown in Table 3, significant differences were found between male and female participants in terms of drop jump and SL-CMJ height (P = 0.001) and drop jump and SL-CMJ flight time (P = 0.001). The remaining variables did not differlyshow significant between the male and female participants. In addition, intra-rater ICC values for movement task values were considered to be good. The values were: drop-jump height (ICC = 0.954), drop-jump flight (ICC = 0.971), drop-jump ground time (ICC = 0.991), drop-jump knee valgus (ICC = 0.937), SL-CMJ height (ICC = 0.979), SL-CMJ flight time (ICC = 0.949), SL-CMJ ground time (ICC = 0.991) and SL-CMJ knee valgus (ICC = 0.994).

**Table 1:** Sociodemographic data of the study population

Data	Total sample (n = 39)	Females (n = 19)	Males (n = 20)	P value females versus males
Age, years	21.20 ± 1.42	20.94± 1.89	21.45 ± 3.28	0.565
Height, m	1.71 ± 0.10	1.65 ± 0.06	1.77 ± 0.10	0.001
Weight, kg	64.84 ± 14.07	58.00 ± 8.32	71.00 ± 15.46	0.002
Body mass index, kg/m^2^	20.3 ± 5.9	19.20 ± 6.40	21.48 ± 5.46	0.236

**Table 2:** Comparison of outcome measurements with and without ankle taping

Measures	No taping	Ankle taping	P value
Drop jump knee valgus F	12.92 ± 6.08	15.73 ± 8.15	0.007
Drop jump height	0.14 ± 0.05	0.13 ± 0.04	0.085
Drop jump flight time	0.33 ± 0.07	0.32 ± 0.05	0.208
Drop jump ground time	0.33 ± 0.05	0.50 ± 0.16	0.476
SL-CMJ knee valgus F	15.25 ± 7.84	13.95± 6.96	0.218
SL-CMJ height	0.13 ± 0.05	0.12 ± 0.04	0.228
SL-CMJ flight time	0.32 ± 0.06	0.31 ± 0.03	0.324
SL-CMJ ground time	0.91 ± 0.30	0.93 ± 0.28	0.762
Y-Balance anterior	86.0 ± 6.77	82.92 ± 7.96	0.001
Y-Balance medial	74.92 ± 9.95	70.56 ± 8.87	0.002
Y-Balance lateral	78.56 ± 7.59	74.82 ± 8.23	0.001
Ankle dorsiflexion DF	47.11 ± 7.90	39.22 ± 5.75	0.001
Ankle DF Asymmetry	7.69 ± 5.65	13.13 ± 7.79	0.001

F = flexion; DF = dominant foot; SL-CMJ = single-leg countermovement jump.

**Table 3:** Comparison of outcome measurements between female and male participants

Measures	Females	Males	P value
	No tape–tape	No tape–tape	No tape–tape
Drop jump knee valgus F	14.11–16.62	11.80–14.88	0.242–0.514
Drop jump height	0.10–0.10	0.17–0.15	0.001–0.001
Drop jump flight time	0.28–0.29	0.37–0.35	0.001–0.001
Drop jump ground time	0.65–0.52	0.47–0.48	0.299–0.501
SL-CMJ knee valgus F	16.09–14.78	14.46–13.17	0.524–0.477
SL-CMJ height	0.10–0.10	0.16–0.14	0.001–0.001
SL-CMJ flight time	0.28–0.29	0.14–0.36	0.001–0.008
SL-CMJ ground time	0.84–0.86	0.98–0.99	0.162–0.524
Y-Balance anterior	84.10–80.10	87.75–85.60	0.098–0.029
Y-Balance medial	71.68–66.68	78.00–4.20	0.046–0.006
Y-Balance lateral	76.42–73.31	80.60–76.25	0.086–0.272
Ankle dorsiflexion DF	48.37–40.34	44.22–38.27	0.108–0.270
Ankle DF Asymmetry	7.08–14.61	8.02–13.81	0.609–0.797

F = flexion; DF = dominant foot; SL- CJM = single-leg countermovement jump.

## DISCUSSION

The purpose of the present study was to assess lower-limb balance and knee biomechanics during landing tasks in participants with AT. There is no doubt that AT protects the ankle joint by preventing extreme movements in plantarflexion. In line with this, the results of the present study showed ROM restriction in both male and female participants in omwhich AT was done, with a mobility decrease of almost 8º. In addition, the yasymmetries between the taping ankle and the free ankle increased by more than 6º. InRomero et al. showed similar values in soccer and basketball players with a decrease in ankle ROM and increase in ankle asymmetry in players with prophylactic AT.^38^ Despite poor evidence of asymmetrical ROM as a risk factor, foot and ankle biomechanics do not cause disturbances in ROM due to external stimuli, such as AT.^39^

Despite the fact that AT has been considered a good prophylactic method for ankle injury prevention, several authors have directly related ankle restriction with knee kinematic alterations.^21^ Klem et al. postulated that an ankle inversion restriction could be related to an increase in the internal rotation of the knee as a compensation mechanism.^40^ The present study showed a significant increase in knee valgus in the frontal plane in the drop-jump task in participants of either sex with a prophylactic AT. However, prior studies have shown that knee compensation movements in the frontal planes occur due to ankle restriction as a result of AT.^1,41^ Previous evidence supports that restriction of ankle dorsiflexion is directly related to knee alterations or a valgus increase in the frontal plane, which is in accordance with the results of the present study.^21,42^ The combination of tibial internal rotation with knee valgus has been described as a knee injury risk factor due to ACL strain.^43^ Both hyperflexion and hyperextension added to internal tibial torque has also been related to the ACL injury mechanism. Therefore, the prevention methods to reduce the internal forces on ACL and internal meniscus during sports activities could help reduce the risk of knee injury.^44^ Thus, based on the results of the present study and previous research, AT should be reconsidered as a prophylactic injury prevention method in healthy participants and among athletes involved in sports which frequently entails jumping and landings. Moreover, AT may also benefit the return-to-play and rehabilitation phases.^38^

In the context of height and flight time values, for both drop-jump and SL-CMJ tasks, we found a slight decrease among participants with AT. Moreover, the drop jump and SL-CMJ ground times were slightly increased in the bandage group. During landing tasks after a drop-jump or SL-CMJ, the joints and lower limbs must be prepared for energy dissipation.^45^ Several authors have suggested that ankle join restriction by AT may interfere with the ability of the lower limbs to attenuate ground reaction forces, which may result in decreasing the performance in jumping tasks, such as drop-jump or SL-CMJ.^7,8,46^ The ability to jump, land and perform effective cutting maneuvers has been associated with better outcomes in sport events and a decrease in the risk of injury among athletes and players who have to be ready for high demands in all the tasks, such as playing basketball or volleyball. Thus, a decrease in these abilities may eincreasing the risk of injury.

In terms of lower limb balance, the present study showed a significant decrease in all three directions of YBT when classic AT was applied. However, several studies have reported the benefits of balance with the use of other ankle bandages, such as kinesiology tape in healthy individuals and athletes.^47,48^ This disparity in results could be explained by the fact that different material properties affect the somatosensory outcomes or provide greater elasticity range.^48^ In this context, disturbances in motor control, poor balance, or lack of neuromuscular aptitudes have been described as predictors of risk of injury in the lower limb. Consequently, all these aspects must be edconsideration before implementation of bracing or AT approaches in healthy individuals.

For complete ankle and foot evaluation, other biomechanical parameters should also be fully assessed, such as leg length discrepancy or mobility of the first metatarsal head.^49,50^

This study had a few limitations. The cross-sectional design of the present study implies that the results should be taken into consideration because only a snapshot of time is considered difficult, making estimation of injury risk in a complete season or period of time an arduous task.^21^ More studies should be performed to assess the effects of AT on foot plantar pressures or to assess the extrinsic and intrinsic foot muscles with electromyography.

### Clinical applications

The results of the present study demonstrate the effectiveness of AT in limiting extreme movements of the ankle joint immediately after its application. However, an increase in knee valgus during landing tasks was observed, which increased the risk of knee injury, such as ACL or meniscus damage. Moreover, a direct negative impact on jump performance was also seen. Therefore, the use of AT is not recommended in healthy individuals. In this regard, we ggessupport that strength or mobility exercises are the best choices for ankle sprain injury prevention in healthy individuals without involving the nearby joints.

## CONCLUSIONS

AT is effective for immediate ankle ROM restriction. However, an increase in knee valgus during drop jump task and a decrease in lower limb balance were observed. Based on these results, AT application in healthy individuals is not recommended due to the increase in injury risk factors.
